# Fabrication of CuO (*p*)–ZnO (*n*) Core–Shell Nanowires and Their *H*_2_-Sensing Properties

**DOI:** 10.3390/ma16134802

**Published:** 2023-07-03

**Authors:** Orhan Sisman, Dario Zappa, Valentin-Adrian Maraloiu, Elisabetta Comini

**Affiliations:** 1 Department of Functional Materials, FunGlass Center, Alexander Dubcek University of Trencin, 91150 Trencin, Slovakia; orhan.sisman@tnuni.sk; 2 Sensor Laboratory, Department of Information Engineering (DII), University of Brescia, Via Valotti 7, 25123 Bresica, Italy; elisabetta.comini@unibs.it; 3 National Institute of Materials Physics, 077125 Magurele, Romania; maraloiu@infim.ro

**Keywords:** CuO–ZnO, metal oxides, core–shell, nanowires, hydrogen sensor

## Abstract

Unlike the conventional one-dimensional (1D) core–shell nanowires (NWs) composed of *p*-type shells and *n*-type cores, in this work, an inverse design is proposed by depositing *n*-type ZnO (shell) layers on the surface of *p*-type CuO (core) NWs, to have a comprehensive understanding of their conductometric gas-sensing kinetics. The surface morphologies of bare and core–shell NWs were investigated by field emission scanning electron microscope (FE-SEM). The ZnO shell layer was presented by overlay images taken by electron dispersive X-ray spectroscopy (EDX) and high-resolution transmission electron microscopy (HRTEM). The pronounced crystalline plane peaks of ZnO were recorded in the compared glancing incident X-ray diffraction (GI-XRD) spectra of CuO and CuO–ZnO core–shell NWs. The ZnO shell layers broaden the absorption curve of CuO NWs in the UV-vis absorption spectra. As a result of the heterostructure formation, the intrinsic *p*-type sensing behavior of CuO NWs towards 250 and 500 ppm of hydrogen (*H*_2_) switched to *n*-type due to the deposition of ZnO shell layers, at 400 °C in dry airflow.

## 1. Introduction

Semiconducting materials have played a significant role in technological progress, providing reliable, tunable, scalable electronic gadgets [1,2]. In addition to the fundamental building blocks of semiconductors like diodes and transistors, they have also been incorporated into other electronic components such as capacitors [3], electromechanical sensors [4], and more [5,6]. Among the diverse range of semiconducting materials, metal oxides (MOXs) have emerged as a popular and expansive branch due to their low cost and easy fabrication processes. At low dimensions, MOXs exhibit unique surface properties that have been useful, particularly in conductometric gas sensor applications. The performance of these sensors is strongly correlated to the characteristics of the integrated MOXs [7,8,9]. Many studies have reported that it could be possible to boost gas sensor performances by combining different MOXs in various forms [10]. In these studies, the band-bending phenomenon at heterojunction points has been reported to explain the superior performances. Due to the different Fermi levels (E_F_) of the two MOXs, electrons flow from higher energies to unoccupied lower-energy states across the interface until the Fermi levels reach equilibrium [11,12,13].

One-dimensional (1D) core–shells have been one of the outstanding heterostructures that are attractive in different applications by conserving the individual characteristics of two metal oxides. For instance, Khan et al. investigated the optoelectronic properties and the new energy band alignments of ZnO–CuO core–shell nanowires for solar energy conversions [14]. Dong et al. reported the higher photocatalytic properties of Co_3_O_4_–ZnO core–shell nanorods compared to bare Co_3_O_4_ nanorods in the decomposition of methylene blue under UV light irradiation [15].

According to the semiconductor nature of MOXs, there are three kinds of heterojunctions possible: *n*-*n*, *p*-*n*, and *p*-*p*. In general, *n*-type MOX materials have been preferred as core (primary) materials for conductometric gas sensor applications due to their higher charge carrier mobility values. Moreover, *p*-type MOXs have been used as shell (secondary) layers to take advantage of their superior catalytic effects. For instance, Arafat et al. demonstrated that TiO_2_–Al_2_O_3_ (*n*-*n*) core–shell nanowires exhibit exceptional performance towards H_2_S, CH_3_OH, and C_2_H_5_OH [16]. Similarly, Fiz et al. showed that the humidity sensing properties of bare SnO_2_ nanowires could be significantly enhanced by fabricating SnO_2_–Nb_2_O_3_ (*n*-*n*) core–shell nanowires using the chemical vapor deposition (CVD) method [17]. Qin et al. observed abnormal *p*-type sensor responses at lower temperatures and remarkable selectivity towards NO_2_ by W_18_O_49_–TiO_2_ (*n*-*n*) core–shell nanowires [18]. In another study, they also revealed that the W_18_O_49_–CuO (*n*-*p*) core–shell nanorods with thinner shell thickness performed better in NO_2_ sensing than the thicker shell layer and bare W_18_O_49_ nanorods [19]. Similarly, Park et al. reported the superior C_2_H_5_OH and NO_2_ sensing properties of *n*-*n* and *n*-*p* one-dimensional heterostructures by fabricating TiO_2_–ZnO [20] and TeO_2_–CuO nanorods, respectively [21].

Recent studies on 1D core–shell nanostructures have shown that an inverse design (*p*-type core/*n*-type shell) can also be effective in the enhancement of gas-sensing properties [22,23,24]. Inspired by this design, we hereby present the fabrication of CuO–ZnO (*p*–*n*) core–shell NWs and their possible application in the chemical sensor field, in particular for *H*_2_ detection. In this study, we synthesized CuO NWs by thermal oxidation and then we deposited a ZnO shell layer by magnetron sputtering on top of CuO NWs, forming a core–shell heterostructure. Hydrogen was selected as the target gas for the preliminary investigation of the chemical sensing performance of the material since it has a small molecule size, which can interact effectively with the core–shell NWs. The combination of the substantial surface area enables the gas-sensing device to achieve a separate control of chemical and physical mechanisms, as demonstrated by this study. With the switching conduction mechanisms, the fundamental structure of semiconductor gas sensors will undergo reconstruction, dividing into two components: carrier conduction and the gas-molecule-trapping and releasing mechanism [25].

## 2. Materials and Methods

### 2.1. Fabrication of CuO–ZnO Core–Shell NWs

Copper (Cu) films, with a thickness of approximately 1 µm, were deposited onto previously cleaned alumina (Al_2_O_3_) substrates (10 mm^2^ × 10 mm^2^) using magnetron sputtering (Kenotec, Binasco, Milan, Italy) in argon plasma with a 75 W applied RF power at a working pressure of 5 mTorr. The substrates were heated up to 200 °C and then cooled down to room temperature before deposition to obtain better adhesion. Following the deposition process, the samples underwent thermal oxidation at 400 °C and atmospheric pressure in a tubular furnace (Lenton Furnaces & Ovens, Hope, UK) for 4 h to synthesize CuO nanowires. To obtain core–shell structures, zinc oxide (ZnO) shell layers (~10–20 nm thickness) were deposited on synthesized CuO nanowires by magnetron sputtering using a ZnO target and argon plasma. The deposition was carried out at 3.5 mTorr working pressure by applying 75 W RF power.

### 2.2. Characterization of CuO and CuO–ZnO NWs

The surface morphologies of the fabricated nanowires were investigated with a field-emission scanning electron microscope (FE-SEM, model LEO 1525, ZEISS, Germany). Microscopic morphological investigations were performed using a JEOL 2100 analytical transmission electron microscope (TEM) (JEOL Ltd., Tokyo, Japan), with LaB_6_ Gun and Tengra CCD (EMSIS GmbH, Munster, Germany), operated at 200 kV in TEM mode. 

The crystalline phases of bare Al_2_O_3_ substrate, CuO nanowires, and CuO–ZnO core–shell nanowires were identified by XRD (Empyrean diffractometer, PANalytical, Almelo, Netherlands) carried out using Cu-LFF (λ = 1.5406 Å) operated at 40 kV–40 mA. Spectrums were recorded by a proportional Xe detector in glancing angle mode (1.5° incident angle). The angular ranges were set to 20°–75° to compare the main crystalline peaks of CuO, ZnO, and Al_2_O_3_. 

The optical properties of CuO NWs and CuO–ZnO core–shell NWs were examined with absorbance measurements carried out with a UV-vis Spectrometer (UV-2600, Shimadzu, Kyoto, Japan).

### 2.3. Conductometric Hydrogen Sensing Measurements

To demonstrate the chemical-sensing performances of the heterostructured material in the presence of *H*_2_, CuO and CuO–ZnO core–shell NWs were transferred onto alumina substrates (2 mm^2^ × 2 mm^2^) using the stamping method. The device configurations were completed by depositing TiW pads and platinum (Pt)-interdigitated (IDT) electrodes on the front side and Pt micro-patterned heaters on the backside of the substrates using magnetron sputtering with the shadow mask technique (with a thickness of 1 μm thickness, an argon plasma, a pressure of 5 mTorr pressure, and a temperature of 300 °C). Then, the sensors were bonded to TO-8 sensor packages with Au wires from both heaters and electrodes utilizing a thermo-sonic bonding machine (4523A Wedge Bonder—Kulicke & Soffa, Willow Grove, Pennsylvania, PA, USA). Conductometric measurements were carried out in the presence of hydrogen (250 ppm and 500 ppm) at 400 °C in dry airflow, with the samples being placed in a climatic test chamber (1 L volume), which was set to 20 °C. The designated working temperature of 400 °C was achieved through the implementation of Pt micro heaters, which were controlled by power supplies (PL330DP, AIM & Thurlby Thandar Instruments, Cambridgeshire, UK). The active layers of the sensors were stimulated by an applied voltage of 5 V, utilizing a power supply (E3631A, Agilent, Santa Clara, CA, USA). The electrical conductance of each sensor was measured using picoammeters (Keithley 486, Tektronics, Beaverton, OR, USA) while injecting hydrogen at concentrations of 250 and 500 ppm. Each injection was allowed to flow within the chamber for a duration of 30 min. Following this, in order to restore the baseline, the airflow was reinstated for a period of 60 min. The performances of sensors were compared by calculating the sensor response values (R) from the variation of conductance values in dry air ($G_{air}$) and hydrogen ($G_{H_{2}}$) flows regarding the conduction type (*n*- or *p*-) as given formulas in Equations (1) and (2).

(1) $$Response\left(R\right)=\frac{G_{air}-G_{H_{2}}}{G_{H_{2}}}(n-type)$$

(2) $$Response\left(R\right)=\frac{G_{H_{2}}-G_{air}}{G_{air}}(p-type)$$

## 3. Results and Discussion

### 3.1. CuO–ZnO Core–Shell NWs

**Figure 1:**
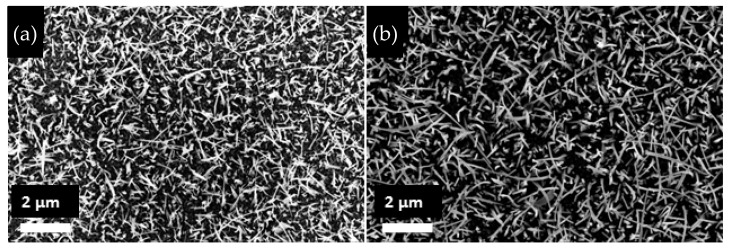
SEM images of (**a**) bare CuO NWs and (**b**) CuO–ZnO core–shell NWs at ×20 k magnification.

The formation of CuO nanowires took place by grain boundary diffusion of Cu^2+^ ions through the surface, which is oxidized by the O^2−^ ions with thermal activation [26,27,28]. It is known that surface tension has an important effect on CuO nanowire density and thickness [29,30]. The alumina had a polycrystalline structure and granular surface. The morphological differences between synthesized pristine CuO NWs and CuO–ZnO core–shell structures can be seen in Figure 1. In both samples, the nanowires were homogeneous through the surface, but they were not identical in size and orientation.

**Figure 2:**
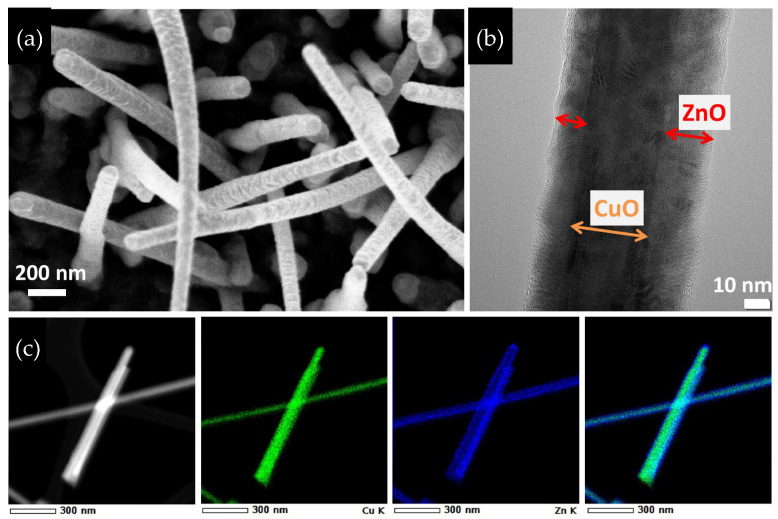
(**a**) FE-SEM, (**b**) HRTEM images of CuO–ZnO core–shell NWs and (**c**) overlay images by EDX measurement: copper in green and zinc in blue colors.

Figure 2a exhibits a detailed SEM image of CuO–ZnO core–shell NWs with a higher magnification. The TEM image in Figure 2b reveals noticeable variations in the thickness of the shell layer. Apparently, the deposition of ZnO layers was not homogeneous on CuO nanowires. The presence of a shadow effect during the ZnO deposition is evident between the left and right sides of the CuO core, likely resulting from the off-axis formation of CuO nanowires. The shadow effect might cause partial coverage by the ZnO layer on some CuO nanowires. Nevertheless, the overlay images obtained through EDX elemental analysis confirmed the core–shell formation of CuO–ZnO nanowires, as given in Figure 2c.

**Figure 3:**
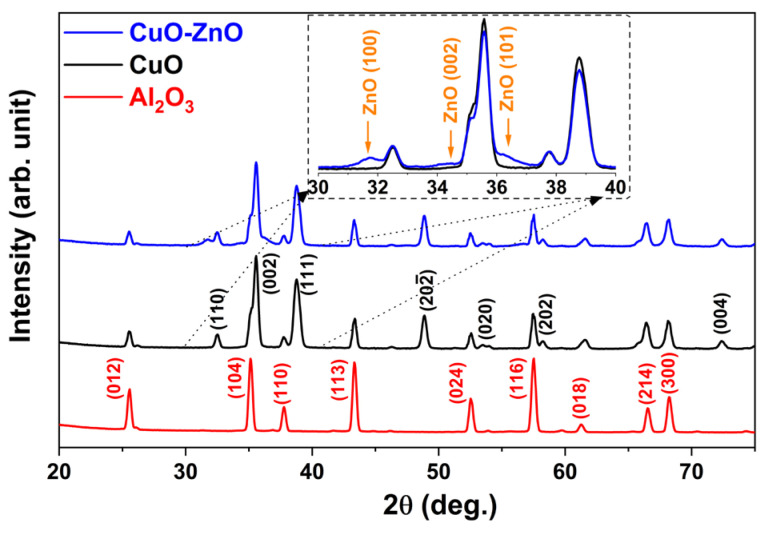
Comparative GI-XRD measurement results.

The investigation of the crystalline structure of heterostructures is crucial for comprehending the gas-sensing mechanism. It is widely acknowledged that dissimilar crystalline phases, even within the same metal oxide, induce distinct surface energies and electrical conduction mechanisms. To disclose the crystalline structure of core–shell nanowires, GI-XRD measurements have been conducted. The comparative GI-XRD spectra belonging to alumina substrate (red line), bare CuO (black line), and CuO–ZnO core–shell nanowires (blue line) are given in Figure 3. The peaks at 2θ angles of 25.6, 35.2, 37.7, 43.0, 52.0, 57.0, 61.0, 66.0 and 68.0 degrees are attributed to (012), (104), (110), (113), (024), (116), (018), (214) and (300) crystalline planes of α-alumina (corundum), respectively [31]. All plane peaks of alumina substrate overlapped with both bare CuO and CuO–ZnO core–shell spectra. 

The CuO crystalline peaks corresponding to (110), (002) and (111) planes were observed at 32.4, 35.8, and 38.3 degrees, respectively, for both bare CuO and CuO–ZnO core–shell samples. Differently, the intensities of these peaks were much lower in the CuO–ZnO measurement profile and there were also small bulges at 31.7°, 34.4°, and 36.2° belonging to the ZnO crystalline planes (100), (002), and (101), respectively, as clearly seen in the inset graph of Figure 3. Considering the small thickness of the ZnO shell layer and shadow effect, the lower intensity and broad profiles of these peaks are expected as the amount of material is low and mostly amorphous. Despite the different deposition methods, similar bulges appeared in the XRD profiles even with a ZnO thickness of 35 nm, as reported by a previous study [24]. In addition, Costas et al. recently reported similar profiles for CuO–ZnO core–shell nanowires [32].

**Figure 4:**
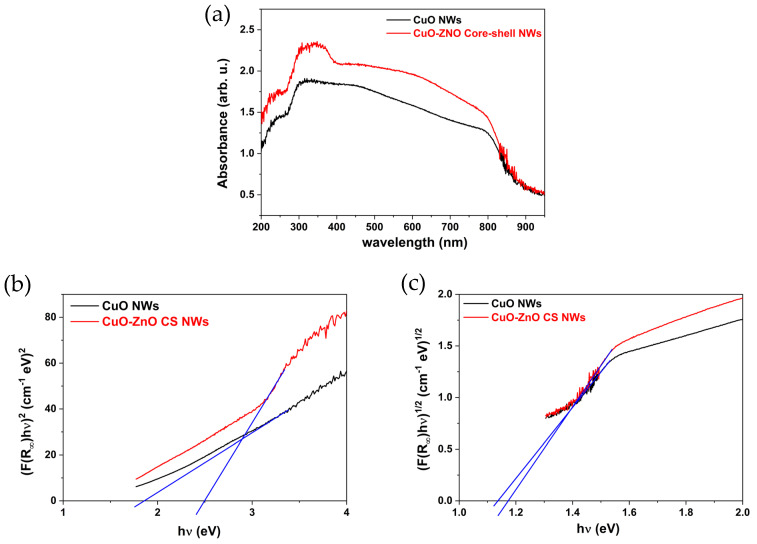
(**a**) UV–vis absorbance spectra, (**b**) direct bandgap energy estimation and (**c**) indirect bandgap energy estimation of CuO and CuO–ZnO core-shell NWs.

The UV–vis absorption profiles shown in Figure 4a were used for a comparative analysis of the optical properties of the CuO and CuO–ZnO core–shell NWs. The results indicated that the core–shell NWs exhibited stronger absorption in the 200–800 nm region compared to bare CuO NWs. The absorption maximum of the CuO NWs sample, around 320 nm, was broadened with the ZnO shell layer. Zhao et al. proposed two probable explanations for the enhanced absorption—the antireflection characteristic of the ZnO shell layers and the enlarged optical absorption area as a result of the core–shell structure [33]. 

The Kubelka–Munk(K-M) function (F($R_{\;\infty \;}$)) [34,35] (Equation (3)) was used to determine the direct bandgap energy values by placing F($R_{\;\infty \;}$) instead of α in the Tauc method (Equation (4)) [36,37].

(3) $$F(R_{\;\infty \;})=\frac{{\left(1-R_{\;\infty \;}\right)}^{2}}{2R_{\;\infty \;}}=\frac{K}{S}$$

(4) $$\left(\alpha {h\nu )}^{2}=B(h\nu -E_{\;g}\right)$$

where $R_{\;\infty \;}$ is the diffusive reflectance of an infinitely thick specimen, while *K* and *S* are the apparent absorption and scattering coefficients, respectively. Due to similar surface structures and not emitting light, the diffuse reflection coefficient *S* does not change appreciably [38]. Therefore, we used approximation K~F($R_{\;\infty \;}$)~α to estimate the optical bandgap energy values of CuO NWs and CuO–ZnO core–shell NWs for direct transitions by plotting $(F({R_{\;\infty \;})h\nu )}^{2}$ vs. hν [39,40]. The calculated curves and estimation lines (Figure 4b) revealed that CuO–ZnO core–shell NWs had an optical bandgap energy value of ~2.5 eV, while CuO NWs had a value of 1.8 eV. Despite the indirect bandgap transition nature of CuO, the energy bandgap value was estimated using the direct transition method. To satisfy the conservation of momentum, indirect optical transitions entail the participation of a photon and, at minimum, one phonon. The energy contribution of the phonon can cause shifts in the indirect bandgap energy value at short wavelengths between 200 and 300 nm [41]. Therefore, we estimated the indirect bandgap energy value as ~1.2 eV for both samples from the absorption decay (corresponds to reflection peak) between 800 and 950 nm, as given in Figure 4c [42]. It is noteworthy that the estimated optical direct bandgap energy value of the CuO NWs was larger than the value of bulk CuO which stood at 1.2 eV. This observation could be attributed to the quantum size effect of nanowires which resulted in the higher bandgap energy value of CuO NWs [43]. Instead, the estimated indirect one fits the reference value well.

**Figure 5:**
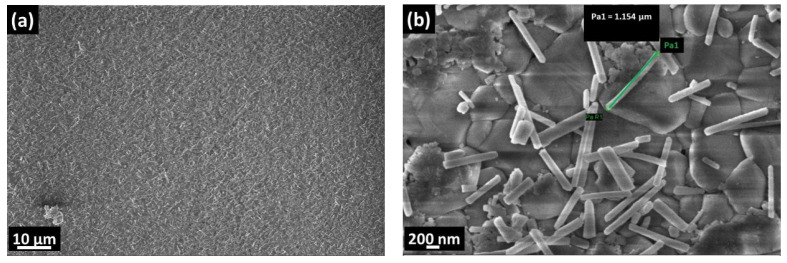
The SEM images of CuO–ZnO core–shell nanowires after the stamping process: (**a**) low-and (**b**) high-magnification images.

The core–shell nanowires were transferred onto an empty alumina substrate using the stamping method to remove the remaining conductive CuO layer under the nanowires. Therefore, the conductance of the stamped core–shell nanowires depended on the junction between unattached nanowires only. As a natural consequence of the stamping method, it was not possible to precisely control the density of nanowires. The transported CuO–ZnO core–shell NWs are reported in Figure 5. The average length of nanowires is around 1 µm (Figure 5b).

### 3.2. Hydrogen Sensing Properties

**Figure 6:**
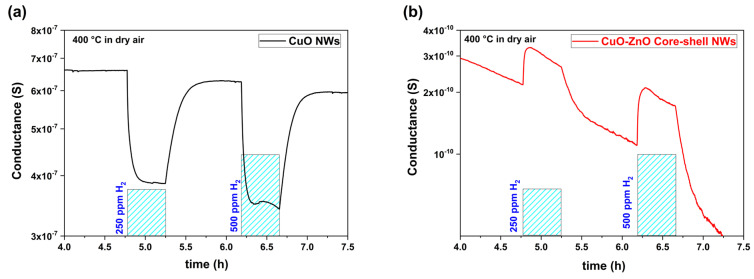
Isothermal dynamic response comparisons (**a**) CuO and (**b**) CuO-ZnO core–shell nanowires at 400 °C in dry airflow.

The isothermal dynamic responses of CuO and CuO–ZnO core–shell NWs towards *H*_2_ are given below (Figure 6). The sensor response values of CuO NWs were calculated as 0.69 and 0.82 towards 250 and 500 ppm of *H*_2_ at 400 °C in dry airflow, respectively. On the other hand, they were calculated as 0.45 and 0.72, respectively, for CuO–ZnO core–shell NWs.

Two different measurement profiles indicated the change in the electrical conduction and sensing mechanism. A classical *p*-type sensing behavior was observed with bare CuO NWs in Figure 6a. The captured electrons, due to oxygen adsorption ($O_{ads}^{-}$) created a hole ($h^{+}$)-accumulation layer on the surface of CuO NWs. During the *H*_2_ exposure, the released electrons reduced the hole ($h^{+}$) concentration in the accumulation layer as given in Equation (5) [28]. Consequently, the electrical conduction value was decreased proportionally to *H*_2_ concentrations.

(5) $$H_{2}+O_{ads}^{-}+h^{+}\to H_{2}O$$

**Figure 7:**
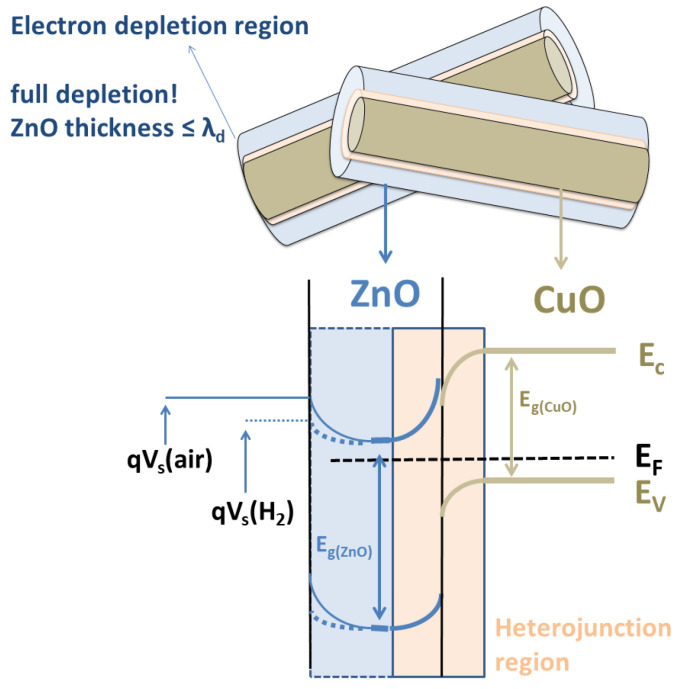
A schematic representation of the energy band diagram of CuO–ZnO core–shell nanowire.

In a study conducted by Katoch et al., it was observed that the conduction type of CuO nanofibers changed from *p*-type to *n*-type upon the deposition of a 5 nm ZnO shell layer. The researchers further proposed that a thicker ZnO shell, exceeding 5 nm, could potentially block gas penetration into the CuO nanofibers [23]. In the present study, however, ZnO shell layers with thicknesses ranging between 10 and 20 nm were utilized. Kim et al. later investigated the ideal thickness of the shell for the gas sensing of CuO–ZnO core–shell nanowires and found that the best sensing performance could be achieved when the thickness of the shell was similar to the Debye length (λ_d_) of ZnO, which was approximately 24 nm at a temperature of 400 °C [24]. Due to the thinner shell thicknesses used in the present study, the ZnO shells were completely depleted of electrons. While the fully depleted shells were found to alter the sensing behavior, they did not enhance the *H*_2_-sensing capabilities. This could be attributed to the electric field smearing effect, which resulted in lower response values [44]. A schematic representation of the band bending at the surface (air/ZnO) and heterojunction region (ZnO/CuO) was provided in Figure 7. The surface band bending occurred at the air/shell interfaces, while another band bending was observed in the heterojunction region between the CuO/ZnO core/shell interfaces. At a temperature of 400 °C, oxygen molecules in the air were adsorbed on the surface of the ZnO shell. In the presence of *H*_2_ gas, the surface potential was reduced due to the released electrons from the adsorbed oxygen ($O_{ads}^{-}$) to the depleted region, leading to an increase in conduction (*n*-type behavior). The chemical reaction that occurred at the surface is given by Equation (6).

(6) $$H_{2}+O_{ads}^{-}\to H_{2}O+e^{-}$$

## 4. Conclusions

An unconventional 1D heterostructured nanowires gas sensor was developed, consisting of a *p*-type CuO core and *n*-type ZnO shell. These *p* (core)–*n* (shell)-designed nanowires were evaluated for their conductometric *H*_2_-sensing capabilities, with a comparative analysis against bare CuO nanowires. The CuO core nanowires were synthesized on alumina substrates via the thermal oxidation of Cu films at 450 °C, while the ZnO shell layers (~10–20 nm) were deposited using magnetron sputtering. Surface analysis measurements were conducted to examine the structural properties of the CuO and ZnO core–shell NWs. Surface morphologies of both bare and core–shell NWs were screened using field-emission scanning electron microscopy (FESEM). The elemental overlay images taken by EDX presented the core–shell structure.

It was observed that the misaligned structure of CuO nanowires resulted in the non-uniform formation of ZnO shell layers, attributed to the shadow effect during magnetron sputtering. The non-uniform shell layer was confirmed by high-resolution transmission electron microscopy (HRTEM) images. Despite the lower amount and variable thickness of the ZnO shell, the characteristic grazing incidence X-ray diffraction (GIXRD) peaks were clearly distinguishable in the comparison profiles of empty alumina substrates, CuO NWs and CuO–ZnO core–shell NW samples. Small bulges in the GIXRD profile of core–shell nanowires indicated the presence of a crystallized ZnO shell layer.

A bandgap modulation was anticipated as a consequence of the *p*-type core (CuO) and *n*-type shell (ZnO) structure. The UV-visible measurements revealed that the presence of the ZnO shell increased the optical bandgap energy value of bare CuO NWs. 

The synthesized and analyzed samples were transferred into blank alumina substrate using the stamping method. The electrical conduction mechanism was reversed from *p*-type to *n*-type due to the deposition of the ZnO shell layer at 400 °C towards hydrogen gas. A possible mechanism for the inverted behavior and lower sensor response values was explained by a fully depleted shell layer and electrical field smearing effect. This opened the possibility of studying, in detail, the switching of semiconducting behavior by operando measurements, providing further details on the role of each material on the gas-trapping and transduction mechanisms.

## Data Availability

The data that support the findings of this study are available from the corresponding author upon reasonable request.
